# Sex and the clock: Exploring sex differences in chronotype and circadian behavior among healthy older adults

**DOI:** 10.1371/journal.pone.0353878

**Published:** 2026-07-16

**Authors:** Natalie S. Pandher, Leslie Yack, Esther Li, Quentin Coppola, Kaitlin B. Cassaletto, Lea T. Grinberg, Thomas C. Neylan, Joel H. Kramer, Christine M. Walsh

**Affiliations:** 1 Edward and Pearl Fein Memory and Aging Center, Department of Neurology, University of California, San Francisco, California, United States of America; 2 Graduate Group in Epidemiology, University of California, Davis, California, United States of America; 3 Stress and Health Research Program, Department of Mental Health, San Francisco Veterans Affairs Medical Center, San Francisco, California, United States of America; 4 Northeastern University, Boston, Massachusetts, United States of America; 5 Global Brain Health Institute, University of California, San Francisco, California, United States of America; 6 Department of Pathology, University of Sao Paulo Medical School, Sao Paulo, Brazil; 7 Department of Pathology, University of California, San Francisco, California, United States of America; 8 Department of Laboratory Medicine and Pathology, Mayo Clinic, Jacksonville, Florida, United States of America; 9 Department of Psychiatry, University of California, San Francisco, California, United States of America; University of Nis Faculty of Medicine: Univerzitet u Nisu Medicinski Fakultet, SERBIA

## Abstract

This study aimed to compare subjective and objective circadian measures with a focus on sex differences and cognition in healthy older adults. A total of 126 participants (aged 65–90 years) completed the Horne & Ostberg Morning-Eveningness Questionnaire (MEQ) and the Circadian Type Inventory (CTI) to assess their morning-evening preference and four circadian traits: rigidity vs flexibility (CTI-FR) and languidity vs vigor (CTI-LV). These self-report measures were compared to actigraphy data from a sub-cohort of 63 individuals who wore wrist actigraphs for 24 hours a day over a 7-day period. Results showed that cognitively healthy older adults tend towards rigidity and vigor (on the CTI) and morningness (on the MEQ). Overall, the languid vs vigorous types and flexible vs rigid types displayed differences in MEQ scores. Sex moderated the association between the CTI-LV and MEQ with a steeper association in males than females (p = 0.012). Actigraphy data showed that males had less stable (interdaily stability, IS, p = 0.03) and more fragmented (interdaily variability, IV, p = 0.001) circadian rhythms than older females. Subjective MEQ scores were strongly predictive of acrophase time (p = 0.009) in males but not females. Using information processing speed (IPS) as a marker of cognitive function, we found that greater circadian rhythm fragmentation (IV) was linked to slower verbal IPS (p = 0.004) in males. Morning preference on the MEQ predicted faster spatial IPS in the overall sample (p < 0.001). These findings provide preliminary evidence of the relationship between subjective sleep preferences and objective circadian data in cognitively healthy older adults with notable sex differences in these relationships.

## Introduction

Maintaining a healthy circadian rhythm is critical for optimal sleep, mood, cognitive function, and overall health. Circadian rhythms are endogenous, 24-hour cycles governing many physiological processes, and the disruption of these rhythms can lead to various homeostatic imbalances [1]. Existing literature has established that circadian rhythms advance and dampen with age [2–4], and these rhythm changes contribute to higher odds of developing dementia or mild cognitive impairment (MCI) [5–8]. Research thus far has emphasized objectively measuring circadian behavior using methods such as wrist-based actigraphy, core body temperature monitoring, and measuring fluctuations in hormonal circadian markers like melatonin and cortisol. Subjective measures of circadian patterns, such as chronotype or sleep schedule preference, have not been studied extensively in the older adult population. We used two subjective measures in this study to provide insight into the qualitative aspects of circadian rhythms which are necessary for a complete and nuanced understanding of circadian behavior in old age. Additionally, we wanted to explore how perceived circadian patterns, and objectively measured ones, relate to each other.

The Morningness-Eveningness Questionnaire (MEQ) is a subjective, chronotype questionnaire that categorizes an individual into a morning type, evening type, or neither type based on their time-of-day preference for doing various activities [9]. Previous research on older adults has explored relationships between age and time of day preference using the MEQ. A study focused on age related differences between a cohort of healthy older adults (mean age: 83 years) matched to an equal number of young controls (mean age: 25 years) found that older adults scored higher on the MEQ (indicating stronger morningness or in other words more morning preference) than their younger counterparts [10]. This finding was supported by the results of a later study done in “middle age” adults (20–59 years of age) in which aging was also associated with an increase in morningness [11]. Morningness has been linked to better subjective health and higher positive affect in old age [12]. Compared to evening types, morning types tend to engage in healthier behaviors, reporting less smoking, fewer sleep disturbances, and higher levels of physical activity [13,14]. Evening preference is positively associated with less daily physical activity and cognitive impairment [15]. None of the literature thus far has found significant sex differences in subjective chronotype assessment.

Another validated, circadian questionnaire widely used is the Circadian Type Inventory (CTI), which measures two dimensions: daytime energy levels (ranging from languid to vigorous) and sleep schedule flexibility (ranging from flexible to rigid) [16,17]. Research using the CTI has been performed predominantly in shift-working populations [18], however, there have been a few landmark studies looking at aging and the constructs of circadian vigor or rigidity. Monk et al. (1991) used the Circadian Type Questionnaire (CTQ), an older version of the current CTI to highlight a positive correlation between aging, greater daytime vigor, and sleep schedule rigidity [10,18]. Marcoen et al. (2015) performed a study on participants aged 18–83 (N = 752) and found a significant sex difference in CTI scores with males demonstrating greater sleep schedule flexibility than females [19]. They also found a negative correlation between flexibility and morning preference, indicating that people with more flexible sleep schedules tend towards evening preference, while those with more rigid schedules tend toward morningness [19]. However, morningness in this study was assessed by the Composite Scale of Morningness [20] which focuses on the degree of morning preference, while the MEQ assesses characteristics like activity and alertness throughout the day to determine chronotype [9]. Sleep schedule rigidity has been linked to better associative memory, independent of sleep time, efficiency, or physical activity levels [21], and consistent sleep schedules have been shown to result in longer sleep duration and better overall health [22].

Of the studies utilizing the MEQ and CTI, only two included objective measures of circadian data [10,11]. Neither of these explored wrist-based actigraphy to assess circadian rhythms, instead using in-lab PSG protocols which have greater precision for measuring sleep but are less ecologically valid for measuring naturalistic sleep-wake rhythms. The existing literature on wrist-based actigraphy and circadian patterns in older adults shows a dampening of rhythm with less overall daytime activity, advanced acrophase times, greater stability (higher interdaily stability), and less fragmented rhythms (lower intradaily variability) [2,4,6,23,24]. In these studies, females generally displayed more robust, stable, and higher amplitude rhythms with less fragmentation when compared to males, however this finding was not restricted to older adults [2,4,24]. Cochrane et al. (2012) used the MEQ in conjunction with wrist actigraphy in an older adult population, but the analyses in this study were limited to comparing circadian parameters between cognitively healthy controls and cognitively impaired individuals [25]. Studies looking specifically at rest-activity rhythm (RAR) in aging populations have focused on the predictive value of circadian measures when it comes to identifying early-stage cognitive impairment and dementia risk. Across the board, decreased amplitude, delayed acrophase, and greater rhythm fragmentation (IV) were associated with higher odds of cognitive decline and greater risk of developing dementia, both in older females and older males [6–8,23,25,26]. The existing circadian literature on older adults highlights the complexity of how age and sex impact biological rhythms and emphasizes the need to consider both subjective and objective measures when studying older populations.

Most research utilizing the MEQ and CTI has focused on younger populations and shift workers, and actigraphy has not been used alongside these subjective circadian questionnaires in older adult populations. This pilot study investigates the relationship between subjective and objective assessments of circadian rhythm in a sample of healthy older adults and how reports of circadian preference and type vary between sexes. We used wrist actigraphy data as a non-invasive, objective method of measuring circadian rhythmicity and the MEQ and CTI as subjective measures. To discern how subjective and objective circadian behaviors might influence cognitive health in older adults, we also looked at information processing speed (IPS) since it has previously been shown to slow with age and is associated with white matter integrity [27,28]. Overall, our goal was to a) characterize the relationships between subjective and objective circadian measures in healthy older adults with an emphasis on sex differences, and b) link the observed circadian behaviors to cognition. Understanding how these circadian patterns are influenced by sex, age, and cognition is essential for developing modifiable interventions to maintain and improve cognitive function in aging populations.

## Methods

### Participants

126 cognitively healthy, community-dwelling, older adults between the ages of 65 and 90 years were enrolled from the Brain Aging Network for Cognitive Health (BrANCH) study at the University of California, San Francisco, Memory and Aging Center (enrollment range: 02/16/2012–07/02/2023). These participants were originally recruited into BrANCH via flyers, newspaper advertisements, and community outreach events. Through BrANCH, all participants underwent a baseline neurological examination, neuropsychological testing, study partner interviews (Clinical Dementia Rating [CDR]), and completed the 15-item Geriatric Depression Scale (GDS) [29,30]. At baseline screening, participants were classified as cognitively unimpaired (CDR = 0) per consensus case conference with a board-certified neurologist and neuropsychologist [31]. Exclusion criteria for the study were severe psychiatric illness, neurologic disorders (e.g., epilepsy, multiple sclerosis), primary sleep disorders (e.g., REM sleep behavior disorder, OSA, narcolepsy), or medical conditions that could impact cognition (e.g., substance use disorders, active chemotherapy). Participants classified as healthy and cognitively unimpaired were then recruited to our study, and they and their partners/informants were asked to provide written, informed consent. Study protocols and consent forms were approved by the University of California, San Francisco Committee on Human Research (IRB #11–07991).

### Questionnaire protocol

To assess morning or evening preference, participants completed the Horne & Ostberg Morningness-Eveningness Questionnaire [9]. The Morningness-Eveningness Questionnaire (MEQ) is a 19-item questionnaire that identifies where along a morning-type to evening-type scale an individual is, based on the times of day during which the participant reports feeling most active and alert. The questions on the MEQ are a combination of Likert-type items and time scales from which participants can choose the ideal span of time during which they would complete various tasks requiring intense focus, physical labor, or nighttime work. In their first validation of the MEQ, Horne and Ostberg (1979) compressed MEQ total scores (range: 16–86) into five categories: definitely morning type (70–86), moderately morning type (59–69), neither type (42–58), moderately evening type (31–41), and definitely evening type (16–30) [9]. However, this categorization did not accurately represent our sample of predominantly morning type individuals with very few definite evening types (N = 2). To address this issue, we collapsed across the moderate and definite evening type groups to form one “evening type”. Thus, analyses were performed for four groups: definitely morning type (70–86), moderately morning type (59–69), neither type (42–58), and evening type (16–41). Our aim in utilizing the MEQ in this study was to understand how it relates to other circadian questionnaires and actigraphy measures.

Participants also completed the 11-item Circadian Type Inventory (CTI), which is a Likert-based questionnaire that assesses an individual’s adaptability to sleep pattern changes by looking at rhythm stability and amplitude [16–18]. Rhythm stability is measured on the Flexible-Rigid (FR) scale where an individual indicates how flexible or rigid they are when faced with sleep schedule changes [16]. Rhythm amplitude is reflected by the Languid-Vigor (LV) scale where an individual reports how languid or vigorous they are in the daytime, especially when faced with reduced sleep [16]. Lower scores on the FR scale are representative of circadian rigidity or a decreased ability to deviate from a set sleep schedule, and higher scores show flexibility, meaning a greater ability to deviate from a set schedule. Similarly, low LV scores mean vigor or higher daytime energy even after a poor night of sleep, and high LV scores are associated with languidness or lethargy following a night of reduced sleep [16,17]. LV scores range from 6 to 30, so we used the midpoint as the cutoff to separate our sample into two groups: languid (LV = 19–30) and vigorous (LV = 6–18). The FR score range is between 5–25, so again using the midpoint, we determined the flexible (FR = 16–25) and rigid groups (FR = 5–15). Our goal in adminstering the CTI was to determine subjective understanding of circadian rhythm stability (FR) and amplitude (LV) in an older population.

### Cognitive function measurement

Cognitive tests assessing processing speed [27,28] were administered within 90 days of the circadian questionnaires and actigraphy measurements in 85 participants. The response latency in seconds was measured for each of the seven visuospatial tasks that made up the testing battery (in the fixed order: Abstract Matching 1, Distance Judgement, Length Judgement, Mental Rotation, Visual Search, Shape Judgement, and Abstract Matching 2). The latency for each task was scaled according to age, combined, and z-scored to create a composite z-score as validated previously in an older adult population [27,28]. Higher z-scores indicate slower processing speed. In addition to this, we used Mini-Mental State Examination (MMSE) scores as a marker of cognitive function in our sample [32]. The MMSE consists of 19 items across 11 domains, including orientation, registration, attention, calculation, recall, naming, repetition, comprehension, writing, and construction [33]. Scores ranging from 25–30 are indicative of normal cognitive status, and we used MMSE < 27 as exclusion criteria to ensure that all participants had baseline normal cognitive function.

### Actigraphy assessment

In a sub-cohort of 63 participants, we recorded at least seven days and nights of actigraphy using the water-resistant Philips Respironics Actiwatch Spectrum Plus (Phillips-Respironics, Bend, Oregon). The watch was worn for up to 24 hours each day, sampled at 32 Hz and processed in 30 second epochs. Participants were asked to briefly remove the watch for showers and baths and to refrain from water sports during the recording period. An actigraphy recording was considered valid if it had no more than four hours of lost data in any twenty-four-hour period, and if there were at least three valid twenty-four-hour periods in the data set. These criteria followed those previously published in older adults [25,34,35]. Data were analyzed using Philips Actiware (Phillips-Respironics, Bend, Oregon) and R packages “mice” [36], “RAR” [37], and “nparACT” [38]. These scripts were used to garner the nonparametric RAR variables used in our analysis. The definitions and derivation of the RAR variables of interest [23,39–41] are presented in Table 1.

**Table 1:** Rest-activity-Rhythm (RAR) variables.

RAR Variable	Definition	Derivation	Interpretation
Interdaily Stability (IS)	measure of the strength of coupling of a rhythm to environmental zeitgebers	ratio between the variance of the average 24-hour pattern around the mean and the overall variance	High IS = stable rhythm Low IS = less stable rhythm
Intradaily Variability (IV)	measure of fragmentation of the circadian rhythm	ratio of hour-to-hour variation relative to total variability	High IV = fragmented rhythm Low IV = intact rhythm
Relative Amplitude (RA)	the difference between activity levels during the most and least active times of day	average intensity of the most active 10 hours (M10) minus the average intensity of the least active 5 hours (L5) divided by M10 + L5	High RA = pronounced rest-activity rhythm Low RA = less pronounced rhythm
Acrophase	time of peak activity occurring during the 24-hour day	time at which the cosinor curve fitted to the circadian rhythm reaches its peak	Before 1:30 pm = advanced phase 1:30 pm to 3:50 pm = normal phase After 3:50 pm = delayed phase

RAR derived by nonparametric analysis of actigraphy data. The specific measures used in this paper include interdaily stability, interdaily variability, relative amplitude, and acrophase.

### Statistical analysis

Data were initially analyzed using IBM SPSS Statistics Version 29.01.00 (171) during 2024−2025. Following revision, all statistical analyses were reproduced in R Version 2025.09.2 + 418 while maintaining consistency with the original analytical approach. Data points three standard deviations from the mean were considered outliers and removed from the dataset. Our variables of interest had kurtosis and skewness values close to zero (within a −2 to +2 range) prior to normalization, so we performed analysis without normalizing. For all regression analyses, normality assumptions were checked by residual plots and Q-Q plots. The Durbin Watson test (d_u_ < d < 4-d_u_) was also performed to rule out autocorrelation as a possible reason for the trends observed. We did not include race, education, physical health, or frailty as covariates to preserve statistical power and because our sample came from a community-dwelling, healthy, generally well-educated, and predominantly Caucasian older adult population with a middle to high socioeconomic status.

For the first part of our research question looking at relationships in the subjective circadian questionnaires, we performed exploratory analysis comparing descriptives between the circadian groups (CTI) and chronotype groups (MEQ) in the entire sample and stratified by sex. To assess baseline differences in the languid-vigorous and flexible-rigid groups, we performed independent samples t-tests for continuous variables (i.e., age, MMSE) and chi-square tests for categorical variables (i.e., retirement status, marital status). We performed similar descriptive analysis to characterize the four time of day preference groups resulting from the MEQ using univariate ANOVA and chi-square tests. To identify sex differences in the association between CTI and MEQ scores, we used a linear regression model covarying for age and sex with interaction terms for sex and circadian type.

For the second part of our research question connecting the subjective measures to RAR and information processing speed, we performed linear regression controlling for age, sex, season, and retirement status. We also included interaction terms for sex here to elucidate sex differences in these associations.

As this study was intended for descriptive and exploratory analysis of subjective and objective circadian measures in a cognitively healthy older adult cohort, adjustments for multiple comparisons were not applied [49,50]. Detailed profiling involving the CTI and MEQ aimed for baseline sample characterization and hypothesis generation rather than formal hypothesis testing. To avoid inflating Type II error rates in this exploratory context, unadjusted p-values < 0.05 were considered statistically significant.

## Results

### Demographics

Participants had a mean age of 74.7 ± 5.4 years and mean MMSE scores of 29.1 ± 1.0 (Table 2). A total of four, female participants took sleep-modifying medications during the study period. These were antidepressants of the following drug classes: tri-cyclic (TCA), selective serotonin reuptake inhibitor (SSRI), and selective serotonin and norepinephrine reuptake inhibitor (SNRI). No significant differences in GDS, MMSE, circadian measures, or information processing speed were observed between medicated and non-medicated participants, and sensitivity analyses excluding these individuals yielded results consistent with the primary analyses. Thus, we retained the four, female individuals in the main analyses to maintain sample size and statistical power. Males and females did not significantly differ across demographic variables (Table 2).

**Table 2:** Demographics.

	Female N = 74*	Male N = 52	Overall N = 126
**Age** (years)			
Mean ± SD (min, max)	73.9 ± 5.8 (65, 90)	75.7 ± 4.7 (67, 88)	74.7 ± 5.4 (65, 90)
**Education** (years)			
Mean ± SD (min, max)	17.3 ± 2 (12, 20)	18.2 ± 2 (12, 21)	17.7 ± 2 (12, 21)
**Marital Status** (at time of study)			
Married/Partnered	43 (58%)	41 (79%)	84 (67%)
Not Married	20 (27%)	9 (17%)	29 (23%)
Unknown	11 (15%)	2 (4%)	13 (10%)
**Retirement Status** (at time of study)			
Retired	38 (52%)	24 (46%)	62 (49%)
Working	12 (16%)	10 (19%)	22 (18%)
Unknown	24 (32%)	18 (35%)	42 (33%)
**Race**			
White	68 (92%)	47 (90%)	115 (91%)
Asian	5 (7%)	2 (4%)	7 (6%)
More than 1 Race	0	1 (2%)	1 (1%)
Other/Unknown	1 (1%)	2 (4%)	3 (2%)
**MMSE**			
Mean ± SD (min, max)	29.2 ± 1 (27, 30)	28.8 ± 1.2 (27, 30)	29 ± 1.1 (27, 30)
**GDS**			
Mean ± SD (min, max)	2.1 ± 2.1 (0, 8)	2.8 ± 3.2 (0, 11)	2.4 ± 2.7 (0, 11)
Missing	19	13	32

*Sample size includes four female participants taking antidepressants during the study period.

### Subjective circadian measures in cognitively healthy older adults

Mean scores on the CTI and MEQ across the sample and within each sex are presented in Table 3. Participants with missing data were excluded from subsequent analysis by listwise deletion. There were no pre-existing sex differences in CTI-LV, CTI-FR, or MEQ scores in this sample.

**Table 3:** Subjective circadian measures.

	Overall N = 126	Female N = 74*	Male N = 52
**CTI-LV**			
Mean ± SD (min, max)	13 ± 4.2 (6,23)	13.5 ± 4 (6,23)	13.6 ± 4.4 (6,23)
Missing	4	3	1
**CTI-FR**			
Mean ± SD (min, max)	12.6 ± 4.2 (5,23)	12.3 ± 4.4 (5,23)	13 ± 3.7 (5,20)
Missing	4	3	1
**MEQ Score**			
Mean ± SD (min, max)	61.8 ± 8.9 (32, 81)	60.8 ± 8.9 (32, 79)	63.2 ± 8.8 (39, 81)
Missing	7	4	3

*Sample size includes four female participants taking antidepressants during the study period.

#### Circadian type inventory.

The healthy older adults in our sample showed a propensity towards rigid sleeping schedules and vigorous dispositions, rather than flexible schedules and languid behavior (S1 Table). Overall, we noted significant differences in MEQ scores between flexible vs rigid individuals (p = 0.01) and also between languid vs vigorous individuals (p = 0.015). In the sex-stratified analysis, this difference remained when comparing flexible males to rigid males (p = 0.048), but not between languid-vigorous males or females (S1 Table). In females, IV was significantly different (p = 0.01) with vigorous women showing more fragmented rhythms than languid women (S1 Table). Additionally, there were some differences in retirement status and marital status (assessed by chi-square test) in both the CTI-FR and CTI-LV subgroups (S1 Table). There were statistically significant differences noted in MMSE scores between the CTI-subgroups in both overall and sex-stratified analysis, but these are likely not clinically meaningful given that MMSE scores were restricted to > 27 to be within the cognitively healthy range.

#### Morningness-eveningness questionnaire.

Most participants reported morning preference or no preference with only four individuals reporting evening preference in this sample. The small number of evening types were excluded from analysis. Comparing across the definitely morning type, moderately morning type, and neither type groups, we found that age (p = 0.011) and acrophase (p < 0.001) were significantly different overall (S2 Table). In the sex-stratified analysis, the acrophase difference remained significant for males (p < 0.001) but not for females (p = 0.238). In females, IS differed between the chronotype groups with the definitely morning types displaying greater rhythm stability (p = 0.042).

### Linking circadian type and time of day preference

**Fig 1:**
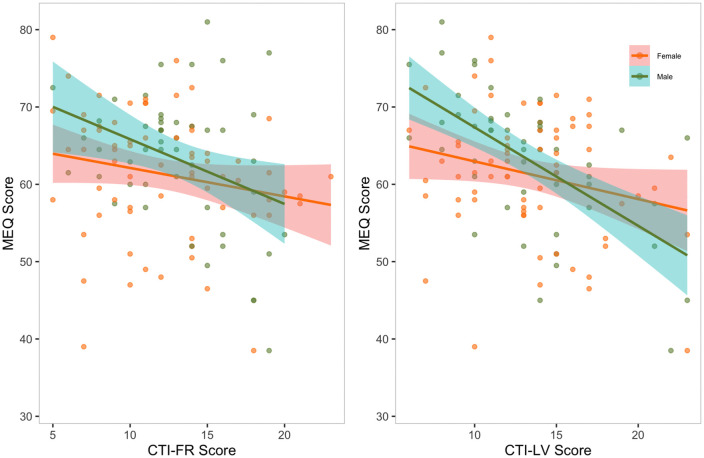
Relationship between circadian type and morningness-eveningness varies between males and females. Circadian languidity, represented by higher CTI-LV scores, is more strongly linked to evening preference, as shown by lower MEQ scores, in males than in females (β = −0.79; p = 0.025; 95% CI: [−1.47, −0.10]; adj R^2^ = 0.23). There is no significant difference in slope between males and females when looking at the association of CTI-FR with MEQ scores.

To further characterize the association between sex, CTI, and MEQ observed in the descriptive analysis above (S1 Table), we used a linear regression model adjusting for age and sex in addition to a second model with an interaction term for sex and CTI to test for sex differences in the association between CTI and MEQ. Significant main effects were observed for sex (β = 12.80; p = 0.009; 95% CI: [3.26, 22.35]) and CTI-LV (β = −0.51; p = 0.032; 95% CI: [−0.97, −0.04]) in the CTI-LV and MEQ regression model which suggest that being male and lower CTI-LV scores are independently associated with higher MEQ scores or morningness. The interaction term for sex and CTI-LV (β = −0.79; p = 0.025; 95% CI: [−1.47, −0.10]) was also significant, indicating that the negative association between CTI-LV and MEQ scores is more pronounced (steeper) in males compared to females (Fig 1). For the regression model for CTI-FR and MEQ, there was a significant main effect of age on MEQ (β = −1.69; p = 0.029; 95% CI: [−3.20, −0.18]), but there was no significant effect of CTI-FR scores or sex.

### Trends in objective circadian data

**Fig 2:**
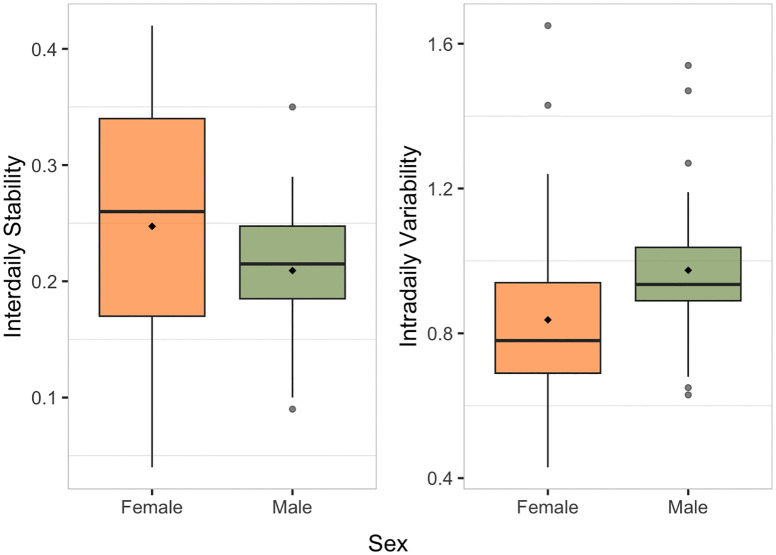
Males exhibit less interdaily stability and more intradaily variability when compared to females in an older adult cohort. Across the participants wearing the Philips Spectrum Plus Actiwatch, there were significant differences in RAR measures of IS and IV between males (green) and females (orange) with males displaying less stable (mean difference = −0.04; p = 0.030) and more fragmented (mean difference = 0.13; p = 0.001) rhythms.

Out of the 126 total participants, 63 individuals recorded actigraphy data by wearing the Philips Spectrum Plus Actiwatch. Rest-activity-rhythm (RAR) measures for all participants with usable actigraphy data are summarized in Table 4, and demographics for this sub-cohort are presented in S3 Table. We computed acrophase in 12-hour clock time by converting radians to time and plotted the spread of acrophase time across our sample. There was a positive correlation between age and acrophase (Pearsons’s r = 0.480, p = 0.001) with acrophase becoming more delayed with age, but there was no link between age and IV, IS, or RA, nor any effect of marital status on these RAR parameters (η^2^ ≤ 0.186). Between the retired and working individuals, IV, IS, and RA were not significantly different, but acrophase time was more delayed in retired participants than in working participants (*t*(28)=2.754, p = 0.010; S1 Fig). We noted significant sex differences in IV and IS with males having less stable (p = 0.030) and more fragmented circadian rhythms (p = 0.001) than females (Fig 2). Overall, the low values for IS and RA and high values for IV align with expected RAR patterns in aging individuals.

**Table 4:** Average Rest-activity-Rhythm in older males and females.

Philips Spectrum Plus Actiwatch Data			
	Female N = 37	Male N = 26	Overall N = 63
**Total Hours of Usable Actigraphy**			
Mean ± SD (min, max)	176.7 ± 36.3 (113.5, 296.7)	175 ± 24.2 (133.8, 260)	176.7 ± 31.7 (113.5, 296.7)
**Total Hours NA**			
Mean ± SD (min, max)	1.6 ± 2.7 (0, 13.2)	1.7 ± 1.7 (0, 6.6)	1.7 ± 2.4 (0, 13.2)
**Interdaily Stability (IS)**			
Mean ± SD (min, max)	0.25 ± 0.10 (0.04,0.42)	0.21 ± 0.06 (0.09,0.35)	0.23 ± 0.09 (0.04,0.42)
**Intradaily Variability (IV)**			
Mean ± SD (min, max)	0.84 ± 0.25 (0.43,1.65)	0.97 ± 0.22 (0.63,1.54)	0.89 ± 0.25 (0.43,1.65)
**Relative Amplitude (RA)**			
Mean ± SD (min, max)	0.56 ± 0.15 (0.2,0.84)	0.54 ± 0.14 (0.26,0.82)	0.56 ± 0.14 (0.2,0.84)
**Acrophase (radians)**			
Mean ± SD (min, max)	14.8 ± 0.96 (12.4,17)	14.8 ± 1.4 (11.9,17.7)	14.8 ± 1.2 (11.9,17.7)
**Acrophase Time (12 hour clock)**			
Mean (min, max)	2:49 (12:23,4:59)	2:49 (11:51,5:39)	2:49 (11:51,5:39)

RAR variables for individuals with actigraphy data recorded using Philips Spectrum Plus Actiwatch.

### Relationships between subjective and objective circadian variables

**Fig 3:**
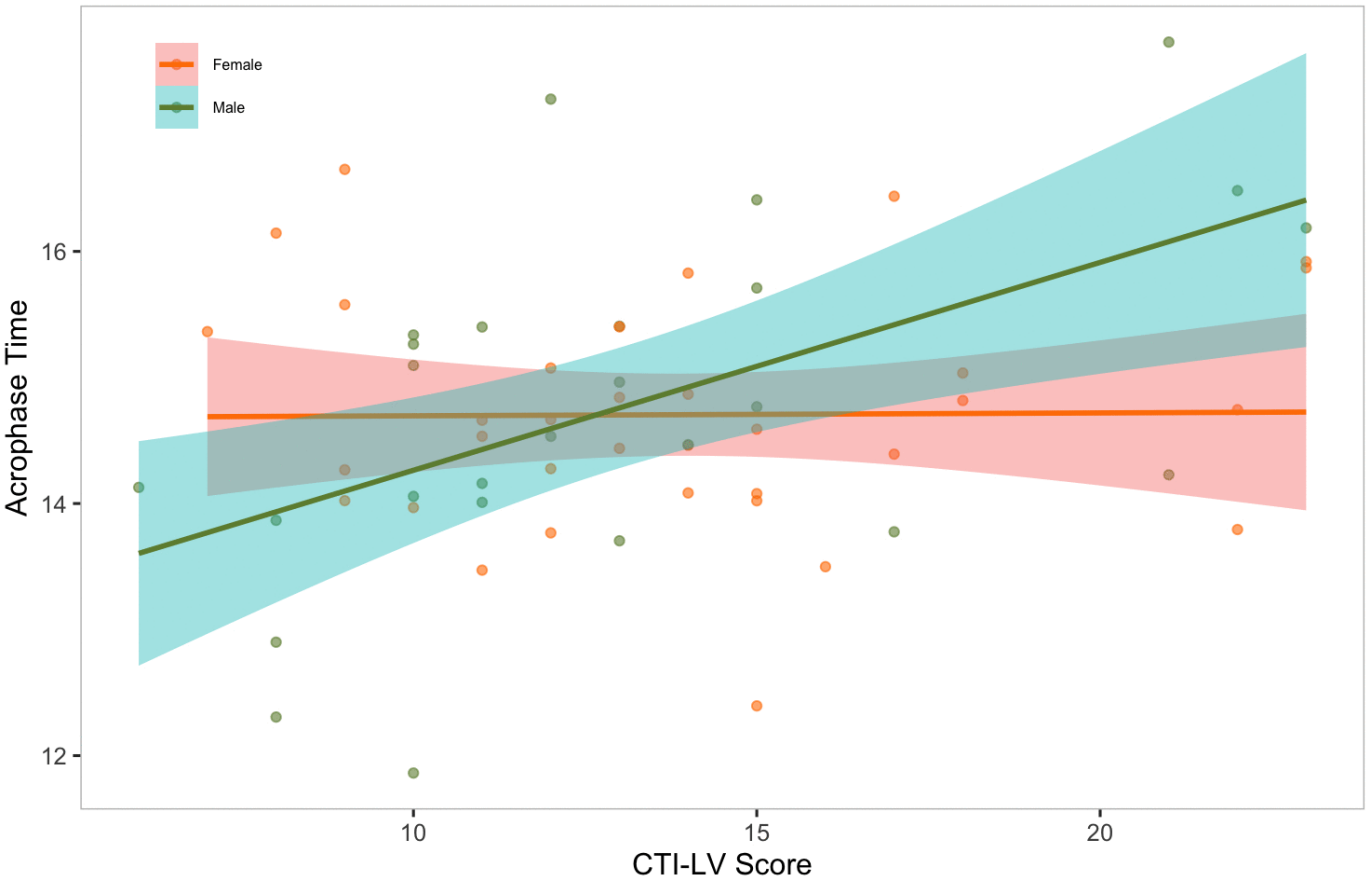
Higher CTI-LV scores associated with delayed acrophase time in males. Males with early acrophase times also had lower CTI-LV scores (β = .151, p = 0.015, 95% CI:[0.03, 0.27]; adj R^2^ = 0.2709) indicating a link between vigorous circadian type and peak activity occurring earlier in the day. However, this relationship was not seen in female participants.

**Fig 4:**
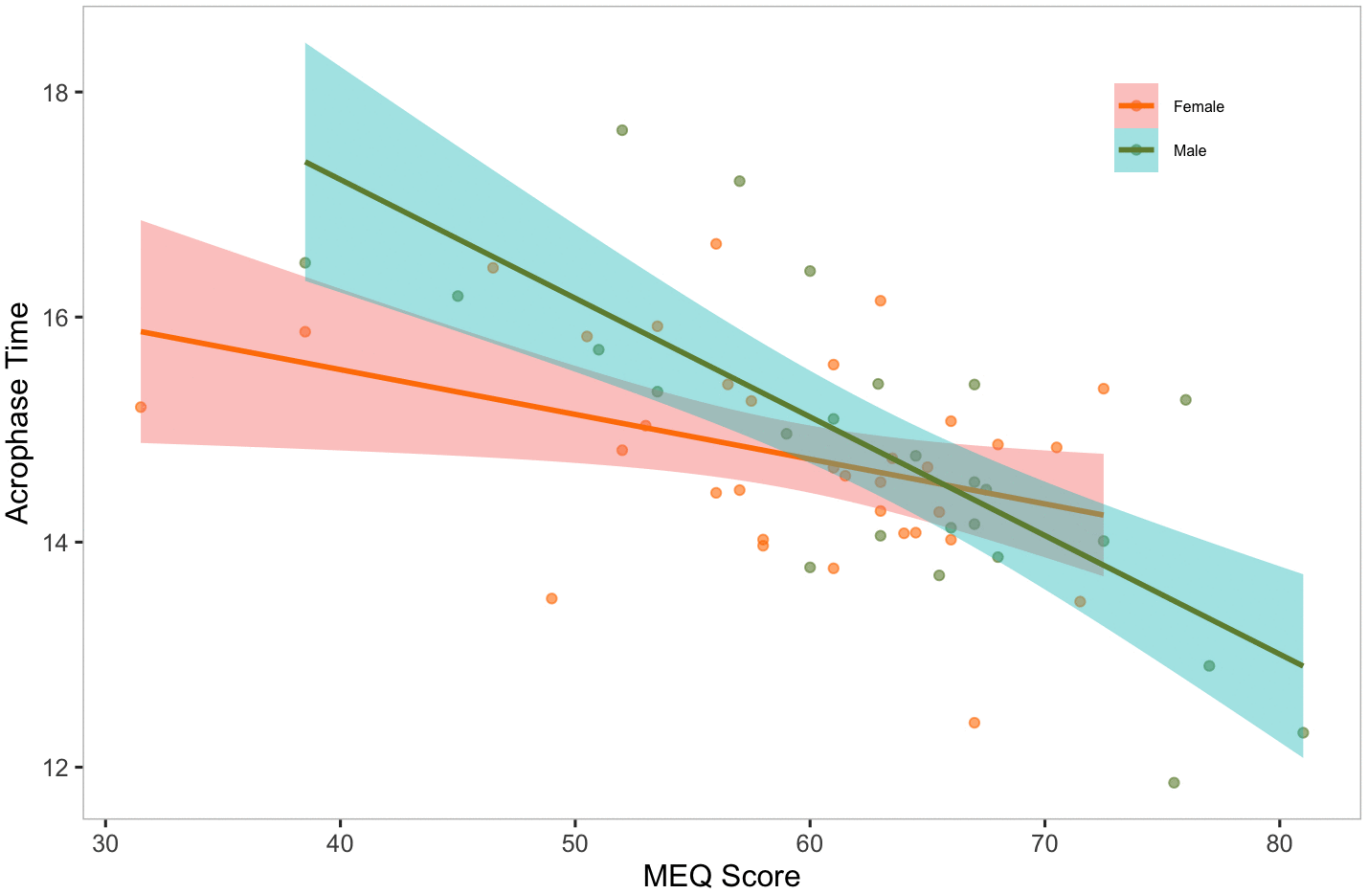
MEQ scores are more negatively correlated to acrophase time in males. Morningness, represented by higher MEQ scores, is associated with earlier acrophase times in males compared to females (β=−.069, p = 0.009, 95% CI:[−0.12, −0.02]; adj R^2^ = 0.399).

Based on the data we collected through actigraphy recordings, we were able to find preliminary evidence for a link between self-reported and objective circadian measures. We investigated, specifically, the relationships between nonparametric RAR variables and the CTI and MEQ. CTI-LV is associated with acrophase (β = 0.086, p = 0.008, 95% CI:[0.02, 0.15]), and sex moderates this association (β = 0.151, p = 0.015, 95% CI:[0.03, 0.27]) with males displaying more delayed acrophase time with increasing languidity than females (Fig 3). There were no significant associations between CTI-LV or CTI-FR and the other nonparametric measures. The relationship between morning preference as measured by the MEQ and acrophase time was also significantly different across sex (β=−0.069, p = 0.009, 95% CI:[−0.12, −0.02]). The slope in males showed a steep negative association (Fig 4) suggesting that subjective morning preference strongly predicted earlier peak activity times in males, while in females this effect was null. The negative association between MEQ scores and acrophase held in a model without an interaction term for sex as well (β = −0.061, p < 0.001, 95% CI:[−0.09, −0.03]). MEQ scores were not a significant predictor for IS, IV, or RA. All of these regression analyses controlled for age, sex, season, and retirement status.

### Cognitive function and morningness-eveningness

**Fig 5:**
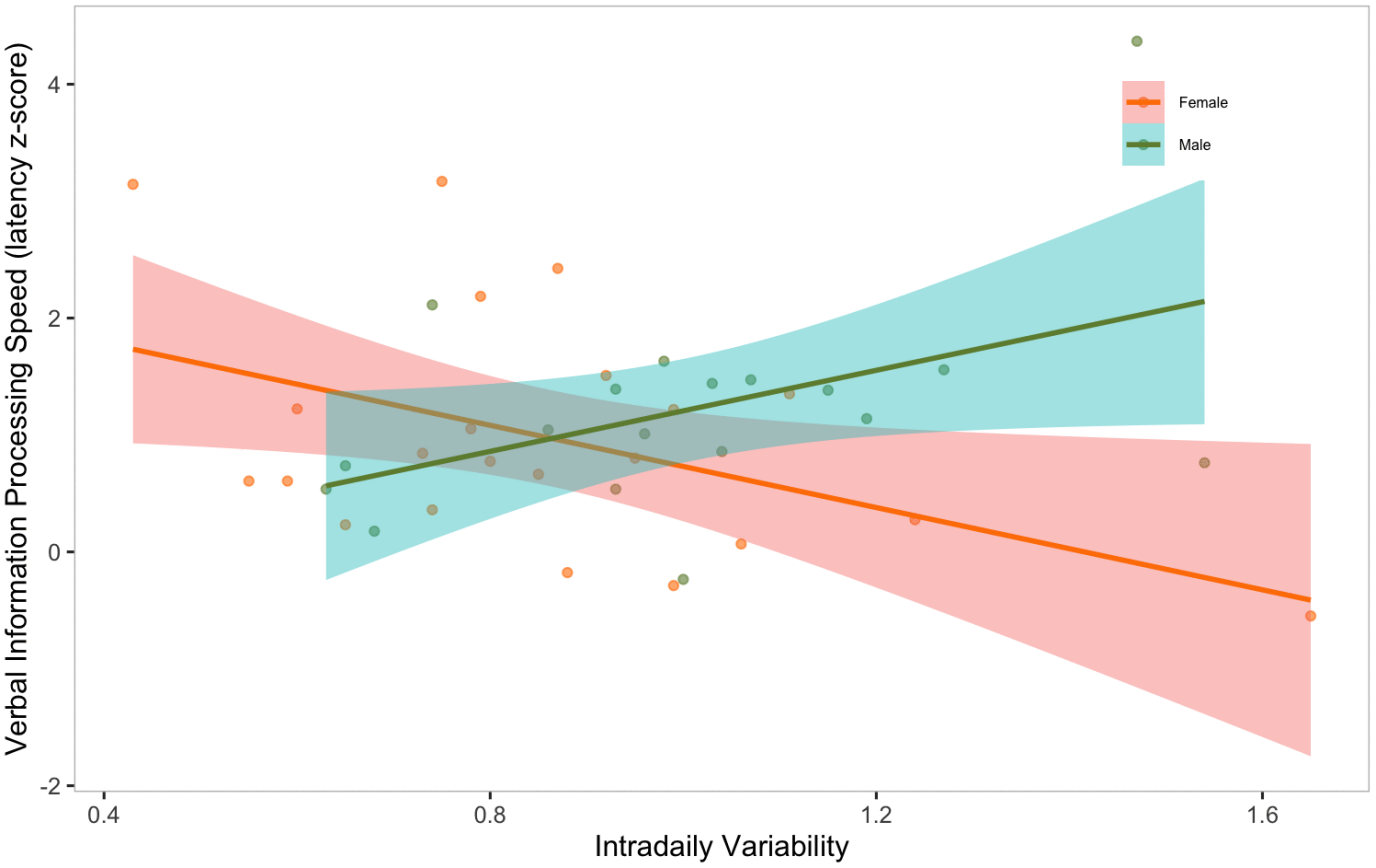
Sex differences exist in the relationship between intradaily variability and verbal information processing speed. The relationship between rhythm fragmentation and verbal IPS latency was significantly different between males and females, as seen by the positive slope in males and negative slope in females (β = 3.25, p = 0.016, 95% CI:[0.65, 5.86]; adj R^2^ = 0.1618).

In this sample average z-scored verbal IPS was 1.2 ± 1 (−0.8, 4.4) and spatial IPS was 2.2 ± 1.1 (−0.09, 5.1). There were no pre-existing sex differences in either verbal or spatial IPS. To contextualize the above findings and explore the possible implications of circadian behavior on cognitive processing, we used a linear regression model to examine the relationship between IPS and the objective and subjective circadian rhythm measures discussed above. CTI scores and MEQ scores were not significant predictors of verbal or spatial IPS in a linear regression model including sex and age. However, we did note a significant negative correlation between MEQ and spatial IPS by a Pearson’s correlation (r = −0.22, p = 0.05, 95% CI: [−0.413, −0.003]). This finding suggests that morning type participants have faster processing speed, or reduced latency, in spatial information tasks, but it is limited in interpretation as we could not control for the time of day at which the processing speed task was administered. We also found that while interdaily variability alone was not a significant predictor for verbal or spatial IPS, there was a significant interaction effect of IV and sex on verbal IPS (β = 3.25, p = 0.016, 95% CI:[0.65, 5.86]). The association of rhythm fragmentation and verbal IPS differed significantly by sex with males showing a steeper increase in latency with greater rhythm fragmentation (high IV) when controlling for age, sex, and seasonal effects (Fig 5). In contrast, females displayed a negative association with verbal IPS latency decreasing with higher IV. We did not control for retirement status here, as IV did not differ significantly working and retired individuals (S1 Fig). IS, RA, or acrophase were not significant predictors for verbal or spatial IPS likely due to the small sample size in the group of participants with both actigraphy and information processing speed measures.

## Discussion

The present study reveals several trends regarding sex differences in circadian behavior and highlights associations between subjective questionnaires, objectively rest-activity rhythm variables, and cognition. Participants were community-dwelling, predominantly Caucasian, married or partnered, and cognitively and physically healthy older adults with an average age of 75 years (Table 2). Consistent with previous studies on healthy older adult populations [10,11,19], most participants displayed rigid sleep schedules and stronger morning preference (Table 3). In the actigraphy sub-cohort, participants had lower interdaily stability (IS), lower relative amplitudes (RA), high intradaily variability (IV), and a mean acrophase time of 2:49 pm (Table 4). These findings align with previously reported circadian patterns of aging, including dampening of rhythm, greater fragmentation, and delayed phase [6,8,23,24,26].

Flexible and rigid individuals and languid and vigorous individuals had significantly different MEQ scores across the overall sample (S1 Table). When stratified by sex, rigid males had greater morning preference than flexible males, and there were no significant differences in MEQ scores between the languid-vigorous or flexible-rigid groups in females. Regression analyses further demonstrated a negative association between circadian languidity and morningness that was significantly stronger in males (Fig 1). Overall, vigorous and rigid males displayed stronger subjective morning preference. The absence of such a relationship in females may reflect greater behavioral flexibility or gender-specific lifestyle factors that weaken the relationship between circadian traits and time-of-day preference [42,43].

Sex differences were also apparent in objective rest-activity rhythm measures. Older males demonstrated less stable (low IS) and more fragmented (high IV) circadian rhythms than older females (Fig 2), a finding consistent with existing literature [2,4,7,8]. We did not find sex differences in RA, despite other studies on older adults noting a decreased amplitude in older males as compared to older females [7,8,26,42]. This may be due to our smaller sample size, better overall health in both sexes sampled in our study, or the fact that we used a non-parametric measure rather than cosinor amplitude. When examining characteristics of the four chronotype groups, acrophase differed significantly across the groups only in males (S2 Table), with definitely morning-type men exhibiting peak activity earlier in the day. In females, rhythm stability (IS) differed across chronotype groups, with definitely morning-type women displaying greater circadian stability than women with less pronounced morning preference (S2 Table). Additionally, individuals reporting more daytime energy (low CTI-LV scores) and stronger morning preference (high MEQ) tended to have earlier acrophase times, suggesting that perceived daytime energy and subjective morningness is predictive of peak activity occurring earlier in the day (Figs 3 and 4). Both these associations were stronger in males than females, further supporting the hypothesis that subjective circadian characteristics may be more tightly coupled to behavioral timing in older males but not in females.

Overall, these findings affirm that self-reported MEQ and CTI answers align with objectively recorded circadian behaviors in older adults and sex differences exist in these relationships. The absence of stronger associations between subjective and objective circadian data in females may be due to circadian and hormonal changes across the menopausal transition, which influence circadian timing. Overnight body temperature and hormone monitoring studies show a shorter intrinsic circadian period in pre-menopausal females along with an advanced phase (earlier core body temperature peaks) and larger amplitude [44,45]. Short circadian period and amplitude have also been shown to remain consistent between pre and post-menopausal females, while subjective morning preference is greater in post-menopausal than pre or peri-menopausal females [44–46]. As mentioned above, gender-related habits such as housework and caregiving that may be more prevalent in female populations, and impact the timing of daily behaviors and sleep patterns, could contribute to the disconnect between subjective circadian traits and objective measures. There may also be an age-related hesitance or conservativeness that reduces reporting of sleep-related complaints in older women as compared to younger women [43]. Lastly, genetic influences on female circadian clock regulation may also contribute to the sex-specific circadian patterns observed in this study. Future research would be needed to determine the exact mechanisms that differentially influence the relationship between these subjective and objective circadian measures in males and females.

The second part of our study connected circadian behaviors to cognitive function in aging adults, and we observed that sex moderated the association between circadian rhythm fragmentation (IV) and information processing speed. Specifically, greater rhythm fragmentation was linked to slower verbal information processing in older males, while the opposite pattern was observed in females. Additionally, we observed a modest correlation between morning preference and faster spatial processing speed, but this finding should be interpreted cautiously, as we were unable to control for the time of day at which cognitive testing occurred and therefore cannot rule out a synchrony effect. Recent literature posits that circadian clock regulation is linked to white matter changes via oligodendrocytes and oligodendrocyte precursor cells (OPCs) [47]. Circadian rhythms may play a role in regulating oligodendrocyte function and myelination, and disruptions in these patterns could contribute to white matter loss [48]. Thus, the association between less fragmented rhythms and faster processing speeds could imply a cognitive benefit from maintaining a stable circadian rhythm in old age. The opposing direction of the relationship between rhythm fragmentation and verbal IPS in males and females further raises the possibility that sex plays an important role in moderating the cognitive consequences of circadian disruption. These hypotheses require further research in a larger sample to determine whether interventions aimed at improving circadian regulation might enhance cognitive performance and delay white matter loss equally in both sexes.

The insights from this study underscore the potential of leveraging circadian traits and chronotype as early indicators for older adults at risk for cognitive decline. Given that subjective questionnaires like the CTI and MEQ align well with objective rest-activity rhythms, these tools have the potential to be included in routine clinical assessments to screen for circadian rhythm disturbances or assess overall circadian health in aging populations. However, the CTI and MEQ have not been used longitudinally to measure circadian change over time or in relation to cognition over time. To better understand how environmental factors, social or behavioral changes, and lifestyle affect CTI and MEQ scores, future research implementing these questionnaires in a longitudinal setting and in relation to other metrics of circadian rhythms, such as core body temperature, would further validate their clinical use. Future studies may also examine whether interventions targeting circadian regulation, such as light exposure, physical activity, or timed melatonin administration, influence the relationship between the CTI and MEQ measures and cognitive outcomes. Additionally, longitudinal investigations focusing on whether the progression from healthy cognition to mild cognitive impairment and eventually to dementia is associated with changes in CTI and MEQ scores would allow for the use of these questionnaires as early methods of identifying at-risk chronotypes and circadian irregularities.

Our findings highlighted significant sex-related differences in circadian behavior and cognitive function, but the underlying mechanisms driving these differences remain unclear. Future research should focus on exploring the hormonal influences, gender-specific lifestyle factors, and genetic mechanisms that could modulate the sex differences identified, beyond that of sex-at-birth itself. The inclusion of neuroimaging techniques (fMRI or tauPET) could provide insight into which brain regions might be affected by circadian behaviors and confirm whether morning/evening preferences and other circadian measures in older adults are linked to neuronal loss or changes in white matter integrity. Limitations of this study include the small sample size, comprised of very active, educated, Caucasian individuals, that reduces the generalizability of our findings. Future research should aim to include larger and more diverse cohorts in terms of physical health, education, socioeconomic status, and race. A written or interview-based questionnaire on lifestyle factors would help further characterize gendered activities/roles and their effect on circadian rhythms in males versus females. Additionally, including measures of environmental and behavioral circadian entrainers, such as light exposure and physical activity, as well as parametric RAR measures, can further contextualize the age and sex related relationship between subjective circadian measures and RAR. Methodologically, the actigraphy data collection relied on a water-resistant, not waterproof, watch, which may have led to the exclusion of water-based periods of activity. Although we attempted to account for this by asking participants to refrain from water sports, the use of a validated, waterproof actigraphy device would be more ideal. Incorporating dim light salivary melatonin assays or core body temperature monitoring alongside actigraphy would also provide a more accurate and comprehensive assessment of circadian rhythms.

## Supporting information

S1 Fig Circadian differences in retired and working older adults show difference in acrophase time but not interdaily stability, intradaily variability, or relative amplitude. In the working individuals, acrophase occurred earlier in the day with an average acrophase time of 14:01, while in retired individuals the average acrophase time was later in the day at 15:09 (mean diff = 1:07, *p* = 0.01). IS (mean diff = 0.047, *p* = 0.205), IV (mean diff = −0.088, *p* = 0.275), and RA (mean diff = 0.08, *p* = 0.132) were not significantly different.
(TIF)

S1 Table Characteristics of languid vs vigorous types and flexible vs rigid types. *p < 0.05; ^**+**^ indicates use of chi-square test rather than independent samples t-test.
(DOCX)

S2 Table Characteristics of time-of-day preference groups categorized by MEQ score. *p < 0.05; ^**+**^ indicates use of chi-square test rather than independent samples t-test. ^1^Evening types excluded from ANOVA due to small cell sizes in that group; ANOVA performed only across 3 chronotype groups; η^2^ denotes effect size.
(DOCX)

S3 Table Additional demographic metrics for sub-cohort with actigraphy data. Demographics for individuals with actigraphy recording using Philips Spectrum Plus Actiwatch.
(DOCX)
