# Supplementary material for: Sex and the clock: Exploring sex differences in chronotype and circadian behavior among healthy older adults
Source: PLoS One. 2026 Jul 16;21(7):e0353878. doi: 10.1371/journal.pone.0353878 (PMC13374977; doi:10.1371/journal.pone.0353878)
Supplement: S1 Table — *p < 0.05; + indicates use of chi-square test rather than independent samples t-test. (DOCX) [file pone.0353878.s002.docx]

**Table S1. Characteristics of Languid vs Vigorous Types and Flexible vs Rigid Types**

| **CTI-LV** | | | | | | | | | | | |
| --- | --- | --- | --- | --- | --- | --- | --- | --- | --- | --- | --- |
|  | **Male** | | | **Female** | | | | | **Overall** | | |
|  | **Languid**  **(n=8)** | **Vigorous**  **(n=43)** | **p-value** | | **Languid**  **(n=7)** | **Vigorous**  **(n=64)** | **p-value** | **Languid**  **(n=15)** | | **Vigorous**  **(n=107)** | **p-value** |
| **Age (years)** | 76.42 | 75.39 | 0.465 | | 73.82 | 73.90 | 0.974 | 75.20 | | 74.50 | 0.625 |
| **Education** | 18.13 | 18.14 | 0.985 | | 17.29 | 17.41 | 0.887 | 17.73 | | 17.70 | 0.953 |
| **Marital Status^+^**  Married  Not Married  Unknown | 7  1  0 | 33  8  2 | 0.736 | | 4  0  3 | 39  18  7 | 0.038* | 11  1  3 | | 72  26  9 | 0.156 |
| **Retirement Status^+^**  Retired  Working  Unknown | 5  1  2 | 19  8  16 | 0.635 | | 3  2  2 | 33  9  22 | 0.602 | 8  3  4 | | 52  17  38 | 0.781 |
| **MMSE** | 28.00 | 29.16 | 0.009* | | 30.00 | 29.18 | <0.001* | 28.44 | | 29.17 | 0.097 |
| **GDS** | 5.00 | 2.29 | 0.218 | | 3.00 | 2.00 | 0.550 | 4.27 | | 2.11 | 0.139 |
| **MEQ** | 54.33 | 64.48 | 0.082 | | 55.17 | 61.92 | 0.121 | 54.75 | | 62.97 | 0.015* |
| **IS** | 0.22 | 0.21 | 0.745 | | 0.20 | 0.25 | 0.273 | 0.21 | | 0.23 | 0.414 |
| **IV** | 0.95 | 0.98 | 0.647 | | 0.71 | 0.86 | 0.010* | 0.83 | | 0.91 | 0.218 |
| **RA** | 0.60 | 0.53 | 0.514 | | 0.54 | 0.56 | 0.878 | 0.57 | | 0.55 | 0.702 |
| **Acrophase** | 16.14 | 14.51 | 0.102 | | 15.08 | 14.65 | 0.473 | 15.61 | | 14.60 | 0.063 |
| **Spatial IPS** | 2.83 | 2.07 | 0.206 | | 1.39 | 2.15 | 0.286 | 2.54 | | 2.12 | 0.396 |
| **Verbal IPS** | 1.49 | 1.10 | 0.254 | | 0.60 | 1.21 | 0.166 | 1.31 | | 1.17 | 0.611 |
| **CTI-FR** | | | | | | | | | | | |
|  | **Male** | | | **Female** | | | | | **Overall** | | |
|  | **Flexible**  **(n=13)** | **Rigid**  **(n=38)** | **p-value** | | **Flexible**  **(n=16)** | **Rigid**  **(n=55)** | **p-value** | **Flexible**  **(n=29)** | | **Rigid**  **(n=93)** | **p-value** |
| **Age (years)** | 75.62 | 75.52 | 0.952 | | 74.74 | 73.65 | 0.547 | 75.13 | | 74.42 | 0.551 |
| **Education** | 17.77 | 18.26 | 0.449 | | 17.44 | 17.38 | 0.929 | 17.59 | | 17.74 | 0.726 |
| **Marital Status^+^**  Married  Not Married  Unknown | 10  3  0 | 30  6  2 | 0.612 | | 8  6  2 | 35  12  8 | 0.445 | 18  9  2 | | 65  18  10 | 0.388 |
| **Retirement Status^+^**  Retired  Working  Unknown | 3  5  5 | 21  4  13 | 0.039* | | 7  1  8 | 29  10  16 | 0.230 | 10  6  13 | | 50  14  29 | 0.193 |
| **MMSE** | 29.67 | 28.61 | 0.001* | | 29.50 | 29.15 | 0.290 | 29.59 | | 28.93 | 0.003* |
| **GDS** | 3.38 | 2.63 | 0.627 | | 1.64 | 2.19 | 0.346 | 2.37 | | 2.37 | 0.998 |
| **MEQ** | 57.65 | 65.25 | 0.048* | | 58.29 | 62.11 | 0.080 | 57.98 | | 63.37 | 0.010* |
| **IS** | 0.23 | 0.20 | 0.245 | | 0.24 | 0.24 | 0.968 | 0.24 | | 0.22 | 0.672 |
| **IV** | 0.97 | 0.98 | 0.976 | | 0.82 | 0.85 | 0.780 | 0.91 | | 0.90 | 0.900 |
| **RA** | 0.57 | 0.53 | 0.429 | | 0.56 | 0.55 | 0.927 | 0.57 | | 0.55 | 0.578 |
| **Acrophase** | 15.14 | 14.56 | 0.272 | | 15.22 | 14.57 | 0.192 | 15.18 | | 14.57 | 0.065 |
| **Spatial IPS** | 2.52 | 2.11 | 0.376 | | 1.95 | 2.15 | 0.500 | 2.26 | | 2.14 | 0.655 |
| **Verbal IPS** | 1.01 | 1.25 | 0.371 | | 1.53 | 1.10 | 0.305 | 1.25 | | 1.16 | 0.725 |

*p<.05

**^+^** indicates use of chi-square test rather than independent samples t-test
