# Supplementary material for: Sex and the clock: Exploring sex differences in chronotype and circadian behavior among healthy older adults
Source: PLoS One. 2026 Jul 16;21(7):e0353878. doi: 10.1371/journal.pone.0353878 (PMC13374977; doi:10.1371/journal.pone.0353878)
Supplement: S2 Table — *p < 0.05; + indicates use of chi-square test rather than independent samples t-test. 1Evening types excluded from ANOVA due to small cell sizes in that group; ANOVA performed only across 3 chronotype groups; η2 denotes effect size. (DOCX) [file pone.0353878.s003.docx]

**Table S2. Characteristics of Time-of-Day Preference Groups Categorized by MEQ Score**

| **MEQ** | | | | | | |
| --- | --- | --- | --- | --- | --- | --- |
| **Male** | | | | | | |
|  | **Definitely**  **Morning-Type**  n = 8 | **Moderately**  **Morning-Type**  n = 29 | **Neither**  **Type**  n = 11 | **Evening**  **Type^1^**  n = 1 | **η^2^** | **p-value** |
| **Age (years)** | 75.4 ± 5.02 | 75.3 ± 4.69 | 76.01 ± 4.92 | 79.75 | 0.004 | 0.914 |
| **Education** | 17.5 ± 2.14 | 18.34 ± 1.93 | 17.73 ± 2 | 20 | 0.03 | 0.467 |
| **Marital Status^+^**  Married  Not Married  Unknown | 5  3  0 | 24  4  1 | 9  1  1 | 0  1  0 | - | 0.412 |
| **Retirement Status^+^**  Retired  Working  Unknown | 2  2  4 | 16  3  10 | 4  3  4 | 1  0  0 | - | 0.448 |
| **MMSE** | 28.8 ± 1.1 | 29 ± 1.14 | 29 ± 1.26 | 29 | 0.01 | 0.940 |
| **GDS** | 1.57 ± 1.72 | 2.95 ± 3.26 | 1.43 ± 2.3 | 11 | 0.06 | 0.350 |
| **IS** | 0.21 ± 0.06 | 0.2 ± 0.07 | 0.21 ± 0.05 | 0.25 | 0.002 | 0.978 |
| **IV** | 0.97 ± 0.32 | 0.96 ± 0.18 | 1.03 ± 0.31 | 0.93 | 0.01 | 0.881 |
| **RA** | 0.54 ± 0.18 | 0.54 ± 0.12 | 0.49 ± 0.1 | 0.64 | 0.02 | 0.807 |
| **Acrophase** | 13.27 ± 1.38 | 14.62 ± 0.77 | 16.42 ± 0.99 | 16.48 | 0.57 | <0.001* |
| **Spatial IPS** | 2.36 ± 1.35 | 1.98 ± 0.81 | 2.51 ± 0.82 | 5.07 | 0.06 | 0.350 |
| **Verbal IPS** | 1.55 ± 1.34 | 1.02 ± 0.5 | 1.21 ± 0.9 | 1.39 | 0.06 | 0.333 |
| **Female** | | | | | | |
|  | **Definitely**  **Morning-Type**  n = 11 | **Moderately**  **Morning-Type**  n = 36 | **Neither**  **Type**  n = 20 | **Evening**  **Type^1^**  n = 3 | **η^2^** | **p-value** |
| **Age (years)** | 72.2 ± 5.23 | 74.28 ± 5.78 | 73.34 ± 5.26 | 84.4 ± 3.81 | 0.02 | 0.533 |
| **Education** | 18.45 ± 1.21 | 17.14 ± 2.11 | 17.05 ± 2.06 | 16.67 ± 2.08 | 0.06 | 0.127 |
| **Marital Status^+^**  Married  Not Married  Unknown | 10  1  0 | 19  12  5 | 12  5  3 | 0  2  1 | - | 0.242 |
| **Retirement Status^+^**  Retired  Working  Unknown | 4  2  5 | 20  6  10 | 12  1  7 | 2  1  0 | - | 0.533 |
| **MMSE** | 29.25 ± 0.71 | 29.06 ± 1 | 29.25 ± 1.06 | 28.5 ± 2.12 | 0.01 | 0.826 |
| **GDS** | 1.44 ± 1.13 | 2 ± 2.09 | 3 ± 2.66 | 1.67 ± 1.53 | 0.07 | 0.203 |
| **IS** | 0.39 ± 0.04 | 0.25 ± 0.12 | 0.22 ± 0.08 | 0.21 ± 0.1 | 0.20 | 0.042* |
| **IV** | 0.76 ± 0.25 | 0.77 ± 0.17 | 0.93 ± 0.34 | 0.84 ± 0.14 | 0.10 | 0.235 |
| **RA** | 0.66 ± 0.07 | 0.54 ± 0.16 | 0.57 ± 0.14 | 0.55 ± 0.23 | 0.06 | 0.394 |
| **Acrophase** | 14.56 ± 0.98 | 14.48 ± 0.82 | 15.06 ± 0.98 | 15.53 ± 0.47 | 0.09 | 0.238 |
| **Spatial IPS** | 1.91 ± 0.95 | 2.07 ± 0.96 | 2.33 ± 1.48 | 3.04 ± 2.92 | 0.02 | 0.681 |
| **Verbal IPS** | 1.13 ± 1.01 | 1.21 ± 1.13 | 1.04 ± 0.99 | 1.13 ± 1.09 | 0.01 | 0.910 |
| Overall | | | | | | |
|  | **Definitely**  **Morning-Type**  n = 19 | **Moderately**  **Morning-Type**  n = 65 | **Neither**  **Type**  n = 31 | **Evening**  **Type^1^**  n = 4 | **η^2^** | **p-value** |
| **Age (years)** | 73.55 ± 5.25 | 74.73 ± 5.31 | 74.29 ± 5.23 | 83.23 ± 3.88 | 0.09 | 0.011* |
| **Education** | 18.05 ± 1.68 | 17.68 ± 2.11 | 17.29 ± 2.04 | 17.5 ± 2.38 | 0.02 | 0.631 |
| **Marital Status^+^**  Married  Not Married  Unknown | 15  4  0 | 43  16  6 | 21  6  4 | 0  3  1 | - | 0.568 |
| **Retirement Status^+^**  Retired  Working  Unknown | 6  4  9 | 36  9  20 | 16  4  11 | 3  1  0 | - | 0.485 |
| **MMSE** | 29.08 ± 0.86 | 29.03 ± 1.06 | 29.17 ± 1.1 | 28.67 ± 1.53 | 0.01 | 0.887 |
| **GDS** | 1.5 ± 1.37 | 2.42 ± 2.68 | 2.48 ± 2.6 | 4 ± 4.83 | 0.04 | 0.337 |
| **IS** | 0.28 ± 0.11 | 0.23 ± 0.1 | 0.22 ± 0.07 | 0.22 ± 0.07 | 0.04 | 0.514 |
| **IV** | 0.89 ± 0.3 | 0.86 ± 0.2 | 0.96 ± 0.33 | 0.87 ± 0.11 | 0.03 | 0.652 |
| **RA** | 0.59 ± 0.15 | 0.54 ± 0.14 | 0.55 ± 0.13 | 0.58 ± 0.17 | 0.02 | 0.797 |
| **Acrophase** | 13.75 ± 1.34 | 14.55 ± 0.79 | 15.44 ± 1.14 | 15.85 ± 0.64 | 0.28 | <0.001* |
| **Spatial IPS** | 2.13 ± 1.15 | 2.03 ± 0.89 | 2.4 ± 1.23 | 3.72 ± 2.37 | 0.09 | 0.060 |
| **Verbal IPS** | 1.34 ± 1.17 | 1.12 ± 0.89 | 1.11 ± 0.93 | 1.22 ± 0.78 | 0.01 | 0.873 |

*p<.05

**^+^** indicates use of chi-square test rather than independent samples t-test

^1^Evening types excluded from ANOVA due to small cell sizes in that group; ANOVA performed only across 3 chronotype groups

η^2^ denotes effect size
