# Supplementary material for: Sex and the clock: Exploring sex differences in chronotype and circadian behavior among healthy older adults
Source: PLoS One. 2026 Jul 16;21(7):e0353878. doi: 10.1371/journal.pone.0353878 (PMC13374977; doi:10.1371/journal.pone.0353878)
Supplement: S3 Table — Demographics for individuals with actigraphy recording using Philips Spectrum Plus Actiwatch. (DOCX) [file pone.0353878.s004.docx]

**Table S3. Additional Demographic Metrics for Sub-Cohort with Actigraphy Data**

| **Philips Spectrum Plus Actiwatch Data** | | | |
| --- | --- | --- | --- |
|  | **Female**  N = 37 | **Male**  N = 26 | **Overall**  N = 63 |
| **Age** (years)  Mean ± SD (min, max) | 73.9 ± 5.6 (65, 90) | 75.4 ± 5 (68, 88) | 74.5 ± 5.4 (65, 90) |
| **Marital Status** (at time of study)  Married  Not Married  Unknown | 20  10  7 | 20  5  1 | 40  15  8 |
| **Retirement Status** (at time of study)  Retired  Working  Unknown | 22  7  8 | 10  7  9 | 32  14  17 |
| **MMSE**  Mean ± SD (min, max)  Missing | 29.1 ± 1.1 (27, 30)  13 | 29.1 ± 1.1 (27, 30)  10 | 29.1 ± 1.1 (27, 30)  23 |
| **GDS**  Mean ± SD (min, max)  Missing | 2.2 ± 2.4 (0, 8)  11 | 3.1 ± 3.5 (0, 11)  9 | 2.5 ± 2.9 (0, 11)  20 |

Demographics for individuals with actigraphy recording using Philips Spectrum Plus Actiwatch.
